# Standardized spider (Arachnida, Araneae) inventory of Hankoniemi, Finland

**DOI:** 10.3897/BDJ.5.e21010

**Published:** 2017-12-18

**Authors:** Pedro Cardoso, Lea Heikkinen, Joel Jalkanen, Minna Kohonen, Matti Leponiemi, Laura Mattila, Joni Ollonen, Jukka-Pekka Ranki, Anni Virolainen, Xuan Zhou, Timo Pajunen

**Affiliations:** 1 Finnish Museum of Natural History, University of Helsinki, Helsinki, Finland; 2 IUCN SSC Spider & Scorpion Specialist Group, Helsinki, Finland; 3 Department of Biosciences, University of Helsinki, Helsinki, Finland; 4 Department of Environmental Sciences, University of Helsinki, Helsinki, Finland

**Keywords:** arthropoda, boreal forest, COBRA, sampling

## Abstract

**Background:**

During a field course on spider taxonomy and ecology at the University of Helsinki, the authors had the opportunity to sample four plots with a dual objective of both teaching on field methods, spider identification and behaviour and uncovering the spider diversity patterns found in the southern coastal forests of Hankoniemi, Finland. As an ultimate goal, this field course intended to contribute to a global project that intends to uncover spider diversity patterns worldwide. With that purpose, a set of standardised methods and procedures was followed that allow the comparability of obtained data with numerous other projects being conducted across all continents.

**New information:**

A total of 104 species and 1997 adults was collected. Of these, 41 species (39%) were Linyphiidae and 13 (12%) Theridiidae. All other families had 6 or less species represented. Linyphiidae were also dominant in terms of adult individuals captured, with 1015 (51%), followed by 428 (21%) Lycosidae, 158 (8%) Tetragnathidae and 145 (7%) Theridiidae. All other families had less than 100 individuals. The most abundant species were *Neriene peltata*, *Alopecosa taeniata*, *Piratula hygrophila* and *Dismodicus elevatus*, all with more than 100 individuals. All sites had between 56 and 62 species and between 445 and 569 individuals.

## Introduction

Dominated by taiga (boreal forest) in the centre and south and tundra in the north, Finland (and the neighbouring Scandinavian Peninsula) marks the transition between the temperate and subarctic zones in Europe. With approximately 45000 multicellular species known to occur in the country (Rassi et al. 2010), Finnish biota is of recent origin, as this area was completely covered by ice during the Last Glacial Maximum until as recently as 10000 years ago. Most species have therefore migrated from the south during the last thousands of years, with very few endemic species constituting exceptions. Most groups show relatively low diversity, both at a local scale (alpha diversity) and when comparing sites on their composition (beta diversity). Low diversity, mainly of endemics and a long tradition of taxonomic work for most groups, means that Finnish fauna and flora are well-known, to the point that Finland currently is the only country worldwide where a full set of organisms, from vascular plants and arthropods to birds and mammals, have already been assessed twice for their threat level according to the International Union for the Conservation of Nature (Rassi et al. 2001, Rassi et al. 2010, Juslén et al. 2013, Juslén et al. 2016).

In Finland and despite obvious knowledge gaps on the distribution of species - the Wallacean Shortfall (Lomolino 2004, Cardoso et al. 2011), spiders are particularly well-known. Seppo Koponen recently described the history of Finnish arachnology (Koponen 2010). The first list of Finnish spider species was published by A. von Nordmann in 1863 with 140 species (von Nordmann 1863). By the beginning of the twentieth century, F. W. Mäklin, K. E. Odenwall and T. H. Järvi had increased the list to 255 species (Mäklin 1874, Odenwall and Järvi 1901, Järvi 1906). Later, major taxonomic and faunistic work directed to spiders has been done mainly by P. Palmgren (e.g. Palmgren 1939, Palmgren 1943, Palmgren 1950, Palmgren 1974a, Palmgren 1974b, Palmgren 1975, Palmgren 1976, Palmgren 1977), P. Lehtinen (e.g. Lehtinen 1964, Lehtinen et al. 1979) and S. Koponen (e.g. Koponen 1977, Koponen 1999, Koponen et al. 2007), with more recent additions by T. Pajunen (e.g. Pajunen et al. 2009, Pajunen and Väisänen 2015) and N. Fritzén (e.g. Fritzén 2005, Fritzén 2012, Fritzén et al. 2015). Currently, this list consists of 647 species (Koponen et al. 2016).

The last arachnological paper by Järvi described the spider fauna around the Tvärminne Zoological Station of the University of Helsinki, in southeast Hankoniemi, listing about 150 species (Järvi 1916). Some decades later, Palmgren dedicated extensive and long-lasting research to this same area, reporting 425 species (Palmgren 1972). This region now has one of the best known spider faunas worldwide. The peninsula of Hanko (Hankoniemi) is the southernmost region of Finland, lying just south of 60 degrees north. Its bedrock is a mixture of Precambrian bedrock and a recent end-moraine complex, running as a continuous ridge from the Russian Karelia through the whole of southern Finland and even further into the northern Baltic Sea. Hankoniemi is therefore dominated by moraine or sandy soils, interspersed with strips of calciferous minerals. The area is comparatively rich, with a number of different biotopes and high species richness for the region (it harbours about 15% of the endangered species of Finland). The main habitat type is pine forest (dominated by *Pinus sylvestris*), often over consolidated dunes fields. Yet, mixed spruce (*Picea abies*) and mixed forests are also very common in smaller areas.

During a field course on spider taxonomy and ecology at the University of Helsinki, the authors had the opportunity to sample four plots with the dual objective of both teaching on field methods, spider identification and behaviour and uncovering the spider diversity patterns found in the southern coastal forests of Hankoniemi. As an ultimate goal, this field course intended to contribute to a global project that intends to uncover spider diversity patterns worldwide (see http://biodiversityresearch.org/research/biogeography/). With that purpose, a set of standardised methods and procedures was followed (Cardoso 2009) that allow the comparability of obtained data with numerous other samples being conducted across all continents. By doing so, these data are guaranteed to be reused for multiple future projects currently being implemented.

## Sampling methods

### Study extent

**Figure 1.**
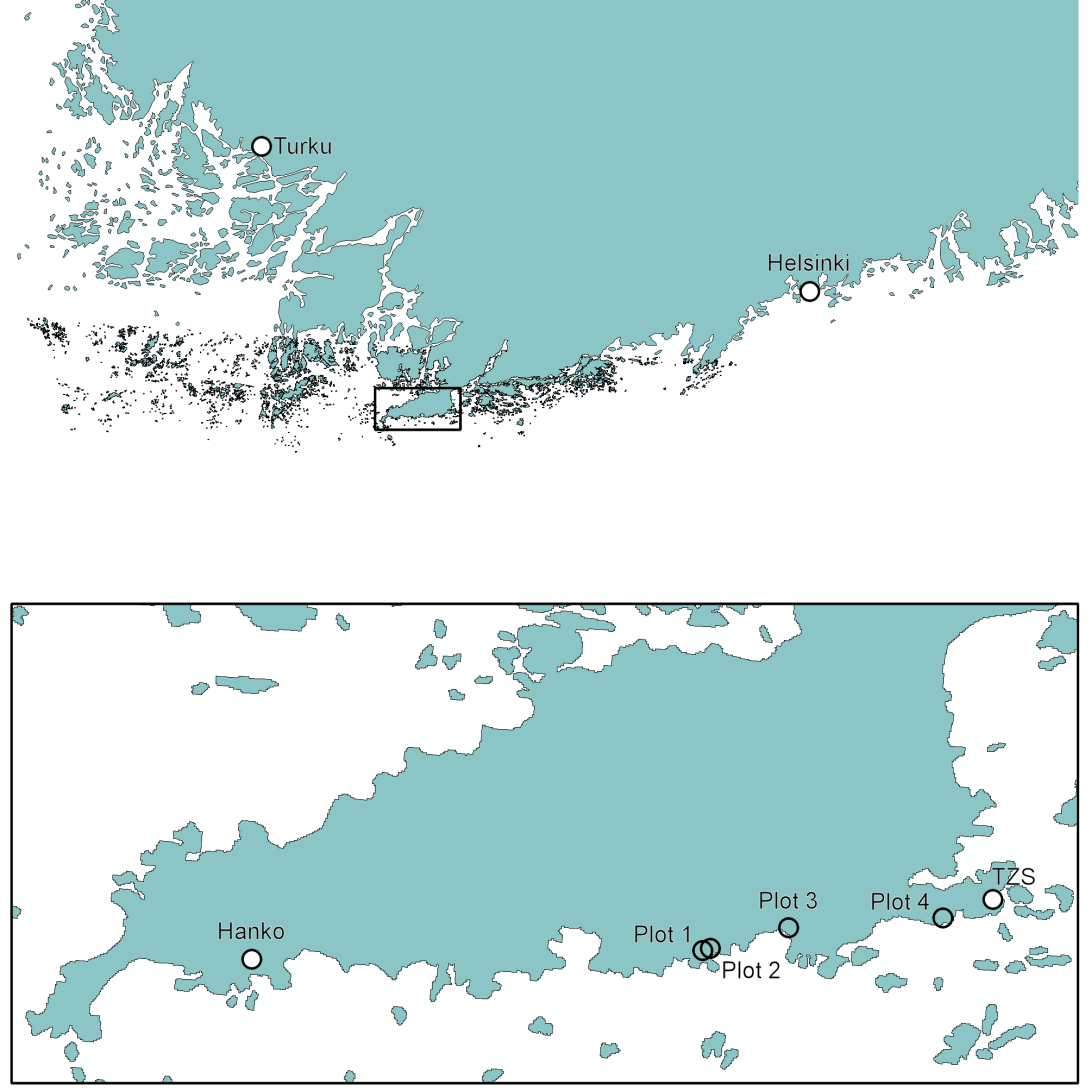
Location of sampling plots in Hankoniemi, southern Finland. TZS = Tvärminne Zoological Station.

Four 50 x 50 m plots following a west to east transect were selected for sampling (Table 1). All were in mixed coastal forests dominated by Norway spruce and Scots pine at sea level (0 - 10 m). Plots 1 and 2 were separated by about 100 m, plot 3 was 1.8 km and plot 4 was 5 km from the first (Fig. 1).

**Study dates**: Samples were collected in June 2016, with all but pitfall trapping being conducted on the 13th and 14th. Pitfall traps were left in the field between the 13th and 26th of June 2016.

### Sampling description

The COBRA - Conservation Oriented Biodiversity Rapid Assessment - protocol at the four plots was followed. This protocol, first proposed for Mediterranean spiders (Cardoso 2009) and very recently adapted for and being applied both on the tropics (Malumbres-Olarte et al. 2016) and islands (Emerson et al. 2017) involves night aerial sampling (4 hours/plot), day/night sweeping (4 hours/plot), day/night beating (4 hours/plot) and pitfall traps (48 traps distributed for 12 samples). In total, it involves about 24 hours of effective work per site (see Cardoso 2009 for details).

## Geographic coverage

### Description

Hankoniemi, Finland

### Coordinates

59.8 and 59.9 Latitude; 23.0 and 23.3 Longitude.

## Taxonomic coverage

### Taxa included

**Table:** 

Rank	Scientific Name	Common Name
order	Araneae	Spiders

## Temporal coverage

**Data range:** 2016-6-13 – 2016-6-26.

## Usage rights

### Use license

Open Data Commons Attribution License

## Data resources

### Data package title

COBRA_Finland_Hankoniemi

### Resource link

http://ipt.pensoft.net/resource?r=cobra_hankoniemi_finland

### Number of data sets

1

## Additional information

**Results**: A total of 104 species and 1997 adults was collected (Table 2, voucher specimens are deposited at the Finnish Museum of Natural History). Of these, 41 species (39%) were Linyphiidae and 13 (12%) Theridiidae. All other families had 6 or less species represented. Linyphiidae were also dominant in terms of adult individuals captured, with 1015 (51%), followed by 428 (21%) Lycosidae, 158 (8%) Tetragnathidae and 145 (7%) Theridiidae. All other families had less than 100 individuals. The most abundant species were *Neriene peltata*, *Alopecosa taeniata*, *Piratula hygrophila* and *Dismodicus elevatus*, all with more than 100 individuals. All sites had between 56 and 62 species and between 445 and 569 individuals.

**Remarks**: The vast majority of species are widespread in Finland and most of Europe. The most notable exception is the uloborid *Hyptiotes paradoxus* (C. L. Koch, 1834), found for the first time on the Finnish mainland, although only represented here by two juveniles. The species was earlier known in Finland only from Ahvenanmaa, an archipelago on the south-western part of the country (Fritzén 2002) and should be a recent addition due to the effects of global warming that drive a northwards movement of many species until recently only recorded south of the country (Fritzén et al. 2015). Additionally, the linyphiid *Entelecara flavipes* was found in Finland just a few years ago in the region of Helsinki, about 100 km east from Hankoniemi (Pajunen and Väisänen 2015). Finally, the thomisid *Diaea dorsata*, which was considered threatened in the 1980s, is now numerous at Hankoniemi.

## Figures and Tables

**Table 1.** Location of sampling plots.

	**Latitude**	**Longitude**
Plot 1	59.83226	23.14042
Plot 2	59.83281	23.14137
Plot 3	59.83779	23.17062
Plot 4	59.84147	23.22869

**Table 2.** Richness and abundance of species per plot (adults only).

**Family**	**Species**	**Plot 1**	**Plot 2**	**Plot 3**	**Plot 4**	**Total**
Anyphaenidae	*Anyphaena accentuata* (Walckenaer, 1802)		1		2	3
Araneidae	*Araneus marmoreus* Clerck, 1757		1			1
Araneidae	*Araneus sturmi* (Hahn, 1831)		3	5	2	10
Araneidae	*Araniella cucurbitina* (Clerck, 1757)		1		2	3
Araneidae	*Cyclosa conica* (Pallas, 1772)	1		1	1	3
Araneidae	*Gibbaranea omoeda* (Thorell, 1870)	1	1		1	3
Araneidae	*Leviellus stroemi* (Thorell, 1870)			1		1
Clubionidae	*Clubiona comta* C. L. Koch, 1839	4	8	5	2	19
Clubionidae	*Clubiona lutescens* Westring, 1851	1			4	5
Clubionidae	*Clubiona subsultans* Thorell, 1875		2	2		4
Dictynidae	*Dictyna arundinacea* (Linnaeus, 1758)		1	4		5
Dictynidae	*Dictyna pusilla* Thorell, 1856	4	4	7	3	18
Dictynidae	*Lathys nielseni* (Schenkel, 1932)		1			1
Gnaphosidae	*Drassyllus praeficus* (L. Koch, 1866)				1	1
Gnaphosidae	*Gnaphosa bicolor* (Hahn, 1833)	1	4	2		7
Gnaphosidae	*Haplodrassus signifer* (C. L. Koch, 1839)			1		1
Gnaphosidae	*Haplodrassus soerenseni* (Strand, 1900)	4	1	3		8
Gnaphosidae	*Haplodrassus umbratilis* (L. Koch, 1866)	1	1			2
Gnaphosidae	*Zelotes clivicola* (L. Koch, 1870)		1			1
Hahniidae	*Cryphoeca silvicola* (C. L. Koch, 1834)	6	12	3	3	24
Linyphiidae	*Agyneta cauta* (O. Pickard-Cambridge, 1902)	7	12	6	1	26
Linyphiidae	*Agyneta ramosa* Jackson, 1912	2	5		10	17
Linyphiidae	*Agyneta subtilis* (O. Pickard-Cambridge, 1863)	1			2	3
Linyphiidae	*Anguliphantes angulipalpis* (Westring, 1851)		1			1
Linyphiidae	*Bathyphantes nigrinus* (Westring, 1851)				1	1
Linyphiidae	*Bathyphantes parvulus* (Westring, 1851)			3	4	7
Linyphiidae	*Ceratinella brevis* (Wider, 1834)		1			1
Linyphiidae	*Dicymbium tibiale* (Blackwall, 1836)	1			1	2
Linyphiidae	*Diplocentria bidentata* (Emerton, 1882)	3				3
Linyphiidae	*Diplocephalus picinus* (Blackwall, 1841)	1	1			2
Linyphiidae	*Diplostyla concolor* (Wider, 1834)		3	1	4	8
Linyphiidae	*Dismodicus elevatus* (C. L. Koch, 1838)	14	36	38	19	107
Linyphiidae	*Entelecara congenera* (O. Pickard-Cambridge, 1879)			1		1
Linyphiidae	*Entelecara erythropus* (Westring, 1851)		8	9		17
Linyphiidae	*Entelecara flavipes* (Blackwall, 1834)		1			1
Linyphiidae	*Gongylidium rufipes* (Linnaeus, 1758)				19	19
Linyphiidae	*Hylyphantes graminicola* (Sundevall, 1830)	4		2	1	7
Linyphiidae	*Hypomma cornutum* (Blackwall, 1833)	3	2		2	7
Linyphiidae	*Macrargus rufus* (Wider, 1834)	1	1			2
Linyphiidae	*Maro minutus* O. Pickard-Cambridge, 1906		1			1
Linyphiidae	*Maso sundevalli* (Westring, 1851)	23	9	32	11	75
Linyphiidae	*Micrargus apertus* (O. Pickard-Cambridge, 1871)	1				1
Linyphiidae	*Minyriolus pusillus* (Wider, 1834)	1	2		1	4
Linyphiidae	*Moebelia penicillata* (Westring, 1851)		2			2
Linyphiidae	*Neriene clathrata* (Sundevall, 1830)	5	11	3	3	22
Linyphiidae	*Neriene peltata* (Wider, 1834)	89	169	88	159	505
Linyphiidae	*Obscuriphantes obscurus* (Blackwall, 1841)	20	12	5	8	45
Linyphiidae	*Oedothorax gibbosus* (Blackwall, 1841)			1		1
Linyphiidae	*Palliduphantes pallidus* (O. Pickard-Cambridge, 1871)			1		1
Linyphiidae	*Pelecopsis elongata* (Wider, 1834)	1	3		1	5
Linyphiidae	*Pityohyphantes phrygianus* (C. L. Koch, 1836)	3	2	2		7
Linyphiidae	*Pocadicnemis pumila* (Blackwall, 1841)	1		1	1	3
Linyphiidae	*Tenuiphantes alacris* (Blackwall, 1853)	3	1	2	15	21
Linyphiidae	*Tenuiphantes cristatus* (Menge, 1866)			1		1
Linyphiidae	*Tenuiphantes tenebricola* (Wider, 1834)	21	16	10	23	70
Linyphiidae	*Walckenaeria antica* (Wider, 1834)			2		2
Linyphiidae	*Walckenaeria atrotibialis* (O. Pickard-Cambridge, 1878)				2	2
Linyphiidae	*Walckenaeria cucullata* (C. L. Koch, 1836)	3	1	4	4	12
Linyphiidae	*Walckenaeria cuspidata* Blackwall, 1833				1	1
Linyphiidae	*Walckenaeria dysderoides* (Wider, 1834)				1	1
Linyphiidae	*Walckenaeria nudipalpis* (Westring, 1851)	1				1
Liocranidae	*Agroeca brunnea* (Blackwall, 1833)		1			1
Lycosidae	*Alopecosa taeniata* (C. L. Koch, 1835)	87	72	61	61	281
Lycosidae	*Pardosa lugubris* (Walckenaer, 1802)	2	4	14	3	23
Lycosidae	*Piratula hygrophila* (Thorell, 1872)	9	3	33	78	123
Lycosidae	*Trochosa terricola* Thorell, 1856			1		1
Mimetidae	*Ero furcata* (Villers, 1789)			1		1
Miturgidae	*Zora nemoralis* (Blackwall, 1861)	3				3
Miturgidae	*Zora spinimana* (Sundevall, 1833)		3	2	1	6
Philodromidae	*Philodromus aureolus* (Clerck, 1757)	3			2	5
Philodromidae	*Philodromus cespitum* (Walckenaer, 1802)	1		1		2
Philodromidae	*Philodromus collinus* C. L. Koch, 1835	5	10	9	6	30
Philodromidae	*Philodromus fuscomarginatus* (De Geer, 1778)	1	1		3	5
Philodromidae	*Philodromus margaritatus* (Clerck, 1757)				1	1
Phrurolithidae	*Phrurolithus festivus* (C. L. Koch, 1835)	1				1
Pisauridae	*Pisaura mirabilis* (Clerck, 1757)			1		1
Salticidae	*Evarcha falcata* (Clerck, 1757)	3	1	12		16
Salticidae	*Neon reticulatus* (Blackwall, 1853)			2		2
Segestriidae	*Segestria senoculata* (Linnaeus, 1758)	2	1			3
Tetragnathidae	*Metellina mengei* (Blackwall, 1869)	30	16	7	29	82
Tetragnathidae	*Metellina merianae* (Scopoli, 1763)	2				2
Tetragnathidae	*Pachygnatha listeri* Sundevall, 1830	6	15	12	12	45
Tetragnathidae	*Tetragnatha dearmata* Thorell, 1873			1		1
Tetragnathidae	*Tetragnatha obtusa* C. L. Koch, 1837	7	7	5	3	22
Tetragnathidae	*Tetragnatha pinicola* L. Koch, 1870	1	1	4		6
Theridiidae	*Enoplognatha ovata* (Clerck, 1757)				4	4
Theridiidae	*Episinus angulatus* (Blackwall, 1836)		1	1	2	4
Theridiidae	*Euryopis flavomaculata* (C. L. Koch, 1836)	1	1	3		5
Theridiidae	*Neottiura bimaculata* (Linnaeus, 1767)			7		7
Theridiidae	*Paidiscura pallens* (Blackwall, 1834)	15	5	4	15	39
Theridiidae	*Parasteatoda lunata* (Clerck, 1757)			2	2	4
Theridiidae	*Phylloneta sisyphia* (Clerck, 1757)			3	1	4
Theridiidae	*Platnickina tincta* (Walckenaer, 1802)	9	9	11	11	40
Theridiidae	*Robertus lividus* (Blackwall, 1836)	9	3	3	1	16
Theridiidae	*Simitidion simile* (C. L. Koch, 1836)			2		2
Theridiidae	*Theridion mystaceum* L. Koch, 1870		2		1	3
Theridiidae	*Theridion pinastri* L. Koch, 1872			1	1	2
Theridiidae	*Theridion varians* Hahn, 1833	3	5	4	3	15
Thomisidae	*Diaea dorsata* (Fabricius, 1777)	10	5	4	10	29
Thomisidae	*Ozyptila atomaria* (Panzer, 1801)			1		1
Thomisidae	*Ozyptila trux* (Blackwall, 1846)		1	8	4	13
Thomisidae	*Xysticus audax* (Schrank, 1803)	1	3			4
Thomisidae	*Xysticus luctuosus* (Blackwall, 1836)		4	1		5
Thomisidae	*Xysticus obscurus* Collett, 1877	2				2
	Species richness	56	62	62	57	104
	Individuals	445	516	467	569	1997
